# Death of Dr. O. K. Parker

**Published:** 1872-12

**Authors:** 


### Editorial.

### Death of Dr. Orlando K. Parker.

RESOLUTIONS OF ERIE COUNTY MEDICAL SOCIETY.

At a special meeting of Erie County Medical Society, convened on account of the death of Dr. O. K. Parker, the president being absent, Dr. T. F. Rochester was invited to preside, who, on taking the chair, made feeling and appropriate remarks, speaking of Dr. Parker as one of the most dignified, intelligent and worthy members of the profession, in whose death both the community in which he lived and the profession sustain irreparable loss.

Dr. MINER said that he had had a most pleasant and intimate professional and social acquaintance with Dr. Parker, and felt his death a personal bereavement. He had long seen that his life was approaching its end, and that his earthly labors must soon cease. Dr. Parker was a physician who enjoyed the love, respect and confidence of the community in a remarkable degree, whose kind manner and warm sympathies won for him a place in the affections of the circle of his acquaintance which but few obtain. As a physician he was intelligent, earnest and capable'; in his relations with the profession courteous, true, honorable, and worthy of the high respect so universally entertained for him. Alas! that one so full of noble impulses should die so soon.

Dr. LAPP had furnished him the following account of his life:

Orlando K. Parker, M. D., of Clarence, Erie county, N. Y., died of consumption Nov. 16, 1872. He was born in the town of Sheldon, Wyoming county, N. Y., August 16, 1826, and was therefore at the time of his death a little more than 46 years of age. After receiving an academical education, he engaged in teaching and clerking until he began studying for the medical profession in the office of Dr. Milton Potter, now of Attica. He graduated at Geneva in the year 1848, and commenced practice at Clarence the same year, where he continued, with the exception of a few months spent as surgeon in the army, until the day of his death. He was married January 18, 1866, to Miss Maria N. Norris, of Buffalo, whom he now leaves a widow with two children. There remains of the family his aged father and an only brother, Dr. L. P. Parker, of Akron, N. Y. By his honorable life and untiring devotion to his professional duties, Dr. Parker won for himself the love and respect of a large circle of friends and the patronage of an extensive community. His death will be widely lamented.

Dr. SAMO made remarks speaking of Dr. Parker as a most dignified, attractive, and capable physician, whose presence in the society added much, and whose death was a great loss.

The following resolutions were unanimously passed:

WHEREAS, Death has removed from our membership Dr. Orlando K. Parker, who has filled during his short but useful life all our offiees of honor and trust, and gained the highest respect and confidence of all,

*Resolved*, That in the death of Dr. Orlando K. Parker we have to mourn the loss of one whose professional life was characterized by most honorable and worthy conduct; who was deeply interested in everything that could promote the welfare or increase the usefulness of our profession, and whose early death is a great loss to this society and the world.

*Resolved*, That we extend to the family and friends of the deceased our deep sympathy in their sad bereavement.

*Resolved*, That we attend the funeral in a body, and wear the usual badge of mourning.

*Resolved*, That these resolutions be published in the Buffalo *Medical and Surgical Journal* and a copy sent to the family of the deceased.

